# Development and Evaluation of a Digital App for Patient Self-Management of Opioid Use Disorder: Usability, Acceptability, and Utility Study

**DOI:** 10.2196/48068

**Published:** 2024-04-01

**Authors:** Van Lewis King Jr, Gregg Siegel, Henry Richard Priesmeyer, Leslie H Siegel, Jennifer S Potter

**Affiliations:** 1 Department of Psychiatry and Behavioral Sciences University of Texas Health Science Center San Antonio San Antonio, TX United States; 2 Biomedical Development Corporation San Antonio, TX United States; 3 Department of Management and Marketing St. Mary's University San Antonio, TX United States

**Keywords:** opioid use disorder, digital health, behavioral medicine, KIOS, mHealth, substance use disorder, substance use treatment, self-management, opioid misuse, substance use, social support, KIOS app, KIOS application, software, patient-centered, opioid

## Abstract

**Background:**

Self-management of opioid use disorder (OUD) is an important component of treatment. Many patients receiving opioid agonist treatment in methadone maintenance treatment settings benefit from counseling treatments to help them improve their recovery skills but have insufficient access to these treatments between clinic appointments. In addition, many addiction medicine clinicians treating patients with OUD in a general medical clinic setting do not have consistent access to counseling referrals for their patients. This can lead to decreases in both treatment retention and overall progress in the patient’s recovery from substance misuse. Digital apps may help to bridge this gap by coaching, supporting, and reinforcing behavioral change that is initiated and directed by their psychosocial and medical providers.

**Objective:**

This study aimed to conduct an acceptability, usability, and utility pilot study of the KIOS app to address these clinical needs.

**Methods:**

We developed a unique, patient-centered computational software system (KIOS; Biomedical Development Corporation) to assist in managing OUD in an outpatient, methadone maintenance clinic setting. KIOS tracks interacting self-reported symptoms (craving, depressed mood, anxiety, irritability, pain, agitation or restlessness, difficulty sleeping, absenteeism, difficulty with usual activities, and conflicts with others) to determine changes in both the trajectory and severity of symptom patterns over time. KIOS then applies a proprietary algorithm to assess the individual’s patterns of symptom interaction in accordance with models previously established by OUD experts. After this analysis, KIOS provides specific behavioral advice addressing the individual’s changing trajectory of symptoms to help the person self-manage their symptoms. The KIOS software also provides analytics on the self-reported data that can be used by patients, clinicians, and researchers to track outcomes.

**Results:**

In a 4-week acceptability, usability (mean System Usability Scale-Modified score 89.5, SD 9.2, maximum of 10.0), and utility (mean KIOS utility questionnaire score 6.32, SD 0.25, maximum of 7.0) pilot study of 15 methadone-maintained participants with OUD, user experience, usability, and software-generated advice received high and positive assessment scores. The KIOS clinical variables closely correlated with craving self-report measures. Therefore, managing these variables with advice generated by the KIOS software could have an impact on craving and ultimately substance use.

**Conclusions:**

KIOS tracks key clinical variables and generates advice specifically relevant to the patient’s current and changing clinical state. Patients in this pilot study assigned high positive values to the KIOS user experience, ease of use, and the appropriateness, relevance, and usefulness of the specific behavioral guidance they received to match their evolving experiences. KIOS may therefore be useful to augment in-person treatment of opioid agonist patients and help fill treatment gaps that currently exist in the continuum of care. A National Institute on Drug Abuse–funded randomized controlled trial of KIOS to augment in-person treatment of patients with OUD is currently being conducted.

## Introduction

The United States is experiencing an opioid epidemic [1]. In 2021, 106,699 people died from overdoses involving opioids, including both prescription and illicit opioids, and the number of deaths continues to increase [1]. Severe opioid use disorder (OUD) is a lifelong and costly illness affecting millions of people worldwide. The socioeconomic impact of OUD affects every aspect of a person’s life; repercussions from long-term opioid use often include legal problems, damage to personal relationships, and significant morbidity and mortality [2].

One of the most effective treatments for severe OUD typically involves opioid agonist treatment [3,4]. While the benefits of medication treatment often occur rapidly, positive behavioral changes for those with more severe OUD can take months and years to develop. Patients receive professional treatment in the clinic, but the struggle with addiction occurs largely in the setting of their everyday lives. OUD treatment often involves periods of exacerbation and remission, and the vulnerability to recurrent drug use remains a life-long risk for many patients. Treatment adherence is often intermittent, and the potential for a recurrence of substance use disorder symptoms carries with it the chronic risk of overdose, trauma, suicide, and infectious diseases [2,5].

Self-management of OUD is a critical component of recovery. Behavioral and supportive therapy used in conjunction with medication is often helpful in changing deeply embedded behaviors that can lead to recurrent drug use [5,6]. However, patients often face a shortage of mental health services, which limits access to care, education, and counseling and impedes their ability to develop effective patient self-management behaviors [7]. Ongoing coaching and feedback are important for recovery for many patients but difficult and expensive to implement, and generally inaccessible during the time of greatest need.

Digital therapeutic software apps are intended to help address this challenge with a variety of approaches being used, and many patients are open to these treatment enhancements [8]. Recognizing the potential of this research and the benefits of evaluating multiple treatment approaches, the National Institutes of Health has increasingly supported digital health behavior research during the past 15-20 years [9,10]. The National Institute on Drug Abuse Clinical Trials Network has conducted several digital health studies examining digital therapeutics for treatment and other technologies for screening, assessment, and a range of other uses [11].

There are growing numbers of increasingly sophisticated apps to address various substance use problems [12-16]. These apps use diverse approaches [17,18], and there is evidence of benefits and cost-effectiveness associated with their use such as improved retention and reduced drug use [18-20].

This study examines the usability, acceptability, and utility of KIOS, a patient-centered computational software system [21]. The KIOS app uses a mobile technology platform to provide daily symptom monitoring and on-demand, individualized feedback. Feedback is based on changes in key treatment variables between successive self-reports to teach and reinforce healthy practices, foster self-management, and promote adherence to treatment plans for patients enrolled in methadone maintenance treatment for OUD. Since engagement with the digital device is key to determining possible efficacy, this study was conducted to make a preliminary evaluation of the KIOS software and client interface in preparation for a randomized controlled trial in a treatment clinic setting.

## Methods

### Overview

This study used a mixed model design to demonstrate technical feasibility; patient engagement; and the acceptability, usability, and utility of KIOS with individuals receiving methadone maintenance for OUD.

### Ethical Considerations

The focus group protocol was approved by BioMed IRB (approval number 18-1-100-OUD). Participants provided informed written consent and were paid for their participation. In addition, the KIOS use study protocol was approved by BioMed IRB and participants provided signed informed consent and were enrolled in a 4-week, single-group, pre-post evaluation of the KIOS app. Participants were paid US $75 after completing orientation and training. They were paid an additional US $75 for participating in a debriefing or focus group. All patient responses were stored in a confidential fashion in a password-protected computer database and only available to approved study personnel. All data were deidentified prior to analysis.

### Developing the User Interface

Contextual design [22], an industry-standard, user-centered methodology, was used to design the user experience. Individuals receiving methadone maintenance were recruited for focus groups at a private treatment clinic (CleanSlate Addiction Treatment Center, n=5) and the local public treatment clinic (The Center for Healthcare Services, n=9). Participants provided informed written consent for their participation. Feedback from participants was solicited about the perceived usefulness of the tool, functionality, benefits, and incentives for using the software.

Based on the focus group feedback, a functional user interface (UI) was created. The web-based KIOS UI includes assessment screens for each of the clinical variables (craving, depressed mood, anxiety, irritability, pain, agitation or restlessness, difficulty sleeping, absenteeism, difficulty with usual activities, and conflicts with others; each rated on a 1-7 scale from none to severe). The determination of the clinical variables is described in the Results section. Inside KIOS, the patient rates the severity of these 10 symptoms and logs self-reported opioid use, alcohol use, and recreational drug use in an important behavior checklist. Participants also reported regular exercise, avoidance of triggers or high-risk situations, and sleep habits (not reported here). The KIOS app analyzes the changes in symptoms during the time between patient self-reports and then delivers advice specific to the patient’s evolving symptoms. On the advice screen of the app, KIOS contextually suggests behaviors that are associated with improving self-report variables, including reaching out to support people or treatment providers, if indicated. In these circumstances, the app encourages the integrated use of comprehensive treatment resources (eg, for increasing depression, patients are directed to consult a medical provider). The results of both the symptom changes and the reporting on important behaviors could also be reviewed by the patient’s individual counselor. This offers the counselor access to real-time data over time on both symptoms and behaviors. It offers the ability to reinforce behavioral treatment planning and maximize the limited counseling time available at each appointment. KIOS also contains a home and menu screen, 6 graphs for tracking symptoms over time, a primary advice screen, a second supplemental advice screen if patients want additional advice, a calendar-based journal, and help screens for technical support, feedback, and emergency contacts for help or crisis situations.

### KIOS Use Study Description

The primary outcome was user satisfaction as measured by acceptability, usability, and utility. Secondary outcomes included self-reporting of drug and alcohol use. The study was conducted exclusively on a web-based interface and via telephone. Participants were recruited from Community Medical Services, a private opioid treatment organization. Staff at Community Medical Services provided potential participants with study information and individuals contacted study staff if they wanted to participate.

Inclusion criteria were (1) interest in study participation; (2) 18 years or older; (3) receiving methadone maintenance for OUD for ≥4 weeks; (4) ability to access KIOS via computer, smartphone, or tablet; and (5) no unmanaged major psychiatric illness or suicidality.

Participants attended a web-based KIOS training session. They were asked to complete KIOS assessments on a web-based interface at least 3 times per week but no more than once daily. KIOS could still be accessed by participants as often as desired to review advice and graphs or access the journal. All assessments were logged on the KIOS server to tabulate the frequency of use.

After the trial, participants completed an 18-question KIOS survey. Participants also completed the System Usability Scale (SUS)-Modified, a single-factor 10-item self-report scale that was used to evaluate participants’ subjective experience using KIOS [23,24].

User data were logged on the KIOS server for the 10 variables from each assessment. At the end of the study, a final web-based debriefing focus group was conducted to gather user feedback not otherwise collected during the study. Participants described their experience in the study and provided their impressions, suggestions, and critiques of KIOS in an open dialogue.

### Analysis

Means, medians, and SDs were calculated for usage, SUS, and patient questionnaire data. KIOS variable scores were averaged over each week for each participant to account for differing numbers of assessments. Calculations were similarly made for an overall KIOS index score (sum of all variables except craving) and for emotional (depressed mood, anxiety, and irritability), physical (pain, agitation or restlessness, and difficulty sleeping), and social (absenteeism, difficulty with usual activities, and conflicts with others) subscales. The craving variable was kept separate due to a consistent body of literature linking it to substance use outcomes [25-27]. The changes in mean values for each week were calculated relative to baseline measures, and 2-tailed Student *t* tests were conducted for significance between baseline and the weekly means. A Pearson correlation was performed by comparing the KIOS index score to the craving scores averaged over the study period.

## Results

### Identifying Key Treatment Variables

Managing recovery from OUD is a complex process characterized by constantly changing intra- and interpersonal circumstances. It was essential to identify a limited number of relevant clinical variables that would both effectively reflect an individual’s subjective behavioral state as well as provide sufficient information to permit the assignment of appropriate behavioral advice. Deidentified data from large-scale randomized OUD clinical trials (the POATS trial: n=653 and the X:BOT trial: n=570) [28,29] were analyzed to provide relevant clinical variables. The KIOS software requires clinical variables that change frequently to operate optimally. Therefore, data obtained from instruments in these studies were analyzed to determine how frequently variables changed from one assessment to the next.

An expert panel (Table 1) selected a subset of 32 variables that were most relevant to OUD recovery. The members of the panel were selected for their expertise in behavioral interventions in OUD and to impart a multicenter perspective to the study. To achieve panel consensus, a modified Delphi process was used [30]. A web-based survey tool and web-based meetings were used to expedite the process. The Delphi process was iterated until consensus was achieved. The decision rule for the inclusion of variables into the system called for the variable to be selected by a majority of the panel members.

**Table 1:** Expert panel.

Panel member	Emphasis	Position
Jennifer S Potter, PhD, MPH	SUD^a^/OUD^b^/pain/CBT^c^	Professor of Psychiatry and Behavioral Sciences, University of Texas Health Science Center, San Antonio
Van L King, MD	SUD/OUD treatment	Professor of Psychiatry and Behavioral Sciences, University of Texas Health Science Center, San Antonio
Roger Weiss, MD	SUD clinical guidelines	Chief, Division of Alcohol and Drug Abuse; Director, Alcohol and Drug Abuse Clinical Research Program, Professor of Psychiatry, Harvard Medical School
Nathanial Katz, MD, MS	Pain/OUD	CEO, Analgesic Solutions, Natick, MA
Mary Velasquez, PhD	SUD/motivational interviewing	Professor and Director of the Health Behavior Research and Training Institute at The University of Texas at Austin Steve Hicks School of Social Work
Erin Finley, PhD, MPH	Evidence-based practices/OUD	Assistant Professor, University of Texas Health Science Center, San Antonio
Kathleen Carroll, PhD	SUD/web-based CBT	Albert E Kent Professor of Psychiatry; Director of Psychosocial Research, Division of Addictions, Yale
Sharon Walsh, PhD	SUD/OUD pharmacotherapies	Professor of Behavioral Science, Psychiatry; Director of the Center on Drug and Alcohol Research, University of Kentucky

^a^SUD: substance use disorder.

^b^OUD: opioid use disorder.

^c^CBT: cognitive behavioral therapy.

Independently, each panel member selected variables with the criteria that a change in the variable is important in determining the patient’s current clinical state. If consensus was not achieved, the panel was queried until the decision rule was met. The variables proposed by the expert panel were then evaluated in a correlation matrix to eliminate redundant variables. Ten individual variables (craving, depressed mood, anxiety, irritability, pain, absenteeism, agitation or restlessness, difficulty sleeping, difficulty with usual activities, and conflicts with others) were selected to capture a person’s clinical state most comprehensively while minimizing the number of questions required of the patient at any assessment.

Next, the panelists proposed combinations of 2 or more variables that provided the most valuable information for describing the current state of the patient and that would be particularly sensitive to identifying transitional worsening or improvement in OUD. This grouping of variables created the underlying structure that mapped the patient trajectory and enabled the development of interventions. Reports were generated by the KIOS software describing all the possible changes for the selected set of interacting variables. The panel supplemented these descriptions with interpretations and recommended interventions.

Appropriate behavioral interventions incorporating cognitive behavioral therapy (CBT) and mindfulness, using the Behavior Change Technique Taxonomy [31] as a reference, were applied to each patient state. These change techniques have been shown effective in multiple studies [32] (Table 2). All recommendations were phrased in patient-friendly language and subjected to a rubric specifying reading level and text length, and that the advice is consistent with a CBT approach. In a previous study, the research team successfully followed a similar approach to chart trajectories and develop self-management interventions for bipolar disorder [21].

**Table 2:** Sample intervention strategy for 4-dimensional variable grouping.

	KIOS logic pathway
Patient state	Anxiety: unchanged Depression: increased Agitation: decreased Difficulty sleeping: increased
Behavioral intervention taxonomy	2.2 Feedback on behavior 2.3 Self-monitoring of behavior 3.1 Social support 11.1 Pharmacological support 12.1 Restructuring the physical environment
Advice	“You’ve reported more difficulty sleeping and a rise in depression. Since these could be related, your first task is to work on sleep hygiene. Consider what you can adjust in your sleep environment to help you rest better at night. Can you reduce noise or light? Start dimming lights earlier in the evening? Do you need earplugs, or do you need to adjust room temperature? Brainstorm what might make you comfortable and restful and try changing up a few things in your sleep environment to help.” “Be sure you’re also following your medication guidelines. If things continue on a downward track, talk to your care provider or someone in your support system. It’s important to pay attention to depressive thoughts and to reach out for help if they don’t resolve.”

### Participants

In total, 19 individuals signed informed consent. Three did not complete orientation. One stopped participating after 11 days and was considered lost to follow-up. The median time in methadone treatment at the beginning of the study was 9 months: 4 patients had been in treatment for ≤6 months, 6 patients had been in treatment for 6-12 months, and 5 had been in treatment for >12 months. Participants (N=19) were 47% (n=9) White (non-Hispanic), 21% (n=4) White (Hispanic), 11% (n=2) more than 1 race, 11% (n=2) American Indian or Alaska Native, 5% (n=1) Black or African American, and 5% (n=1) Asian. The mean age of all participants was 34.4 (SD 7.3) years, and 63% (n=12) were women.

### KIOS Use and Acceptability

In total, 15 of 16 participants who logged in to KIOS at least once finished the trial. Those 15 participants completed 191 total assessments, averaging 12.7 (SD 3.8) per user (median 13, IQR 11-14). During the poststudy debriefings, several participants requested continued access to KIOS after the trial ended without further compensation; 6 participants used KIOS poststudy and 1 participant used the app for over 10 months.

The final debriefing focus group generated many comments that described the individualized experience of using the app. All participants who attended the group stated it was helpful and worth using. For example, focus group attendees noted that KIOS gave timely and responsive advice, helped with adherence to treatment goals, and promoted reflection and motivation. Some commented on how natural the advice seemed, almost like a counselor was responding in real time.

Some participants mentioned features that could improve KIOS such as a customized reminder system to specify the time of day to use the app, features to connect directly with counseling staff or peer support, gamified content (such as daily challenges, more content that included pictures), positive reinforcement (such as digital trophies, badges, or monetary rewards), and expanded and more specific description of exercise or meditation practices beyond those currently included in the app.

### KIOS Usability

The mean SUS-Modified score was 89.5 (SD 9.2; median 92.5, IQR 85-95). Higher scores indicate greater usability. The psychometric properties of the SUS have been validated and replicated, and the score for KIOS falls slightly below the top score, “best imaginable=90.9” and above “excellent=85.5” on an adjective rating of the SUS [23,24] (Table 3).

**Table 3:** System Usability Scale-Modified scores (1=strongly disagree to 5=strongly agree).

	Raw score, mean (SD)
I think I would like to use this system frequently	4.40 (0.74)
I found the system unnecessarily complex	1.40 (0.83)
I thought the system was easy to use	4.93 (0.26)
I think that I would need the support of a technical person to be able to use this system	1.13 (0.52)
I found the various functions in this system were well integrated	4.40 (0.83)
I thought there was too much inconsistency in this system	1.20 (0.56)
I would imagine that most people would learn to use this system very quickly	4.87 0.35)
I found the system very cumbersome to use	2.40 (1.35)
I felt very confident using the system	4.80 (0.41)
I needed to learn a lot of things before I could get going with this system	1.47 (0.74)

### KIOS Utility

The KIOS questionnaire was developed by the research team to evaluate the utility of the KIOS app. The mean response to all KIOS questionnaire data was 6.32 out of a maximum of 7.0 (SD 0.25), indicating an overall strong agreement with most statements. The highest rated statement was “I see benefits using KIOS,” which averaged 6.73 out of 7 (SD 0.46); each respondent rated this item as either 6 or 7. The lowest rated item was “KIOS helped identify some personal triggers,” which was still generally well agreed upon and rated 5.73 (SD 1.62; Table 4).

**Table 4:** KIOS satisfaction survey (1=disagree to 7=agree).

KIOS satisfaction survey question	Value, mean (SD)
I was able to read and understand the advice	6.67 (0.62)
The advice felt personal and fitting to how I was feeling	6.20 (1.08)
I found the advice helpful	6.33 (0.98)
I learned something new from the advice	6.33 (0.98)
I liked the tone and style of the written advice	6.67 (0.62)
The length of the advice was appropriate	6.53 (0.83)
Overall, I found the graphs useful	6.33 (0.98)
Overall, the graphs were easy to understand	6.47 (1.13)
Overall, the graphs were visually appealing	6.13 (1.06)
The important behaviors checklist is helpful	6.60 (0.63)
I see benefits from using KIOS	6.73 (0.46)
Using KIOS improved my self-management skills	6.13 (0.74)
KIOS helped me to better understand my recovery process	6.20 (0.77)
Using KIOS helped me to become more aware of subtle shifts in my physical, mental, and social health	6.47 (0.64)
Using KIOS helped me to become more aware of practices that can restore or maintain balance in my physical, mental, and social health	6.47 (0.83)
Using KIOS helped me to identify some personal triggers	5.73 (1.62)
Using KIOS increased my overall satisfaction with my health status	5.93 (1.10)
Using KIOS adds value beyond the treatment that I typically receive	6.13 (0.92)
All questions	6.34 (0.25)

### KIOS Assessment Data

The Healthy Behavior Checklist allows patients to check yes or no for engaging in the behavior at each assessment. The behaviors include opioid use, recreational drug use, alcohol use, exercising, good sleep habits, and avoiding triggers and high-risk situations. Only 1 participant self-reported 1 occurrence of unprescribed opioid use. This occurred at the baseline assessment, and no other illicit opioid use was reported by any participant during the study. Four participants reported 18 occurrences of alcohol use and 3 others reported 24 instances of recreational drug use (nonspecified); no participant reported both alcohol and recreational drug use.

**Figure 1:**
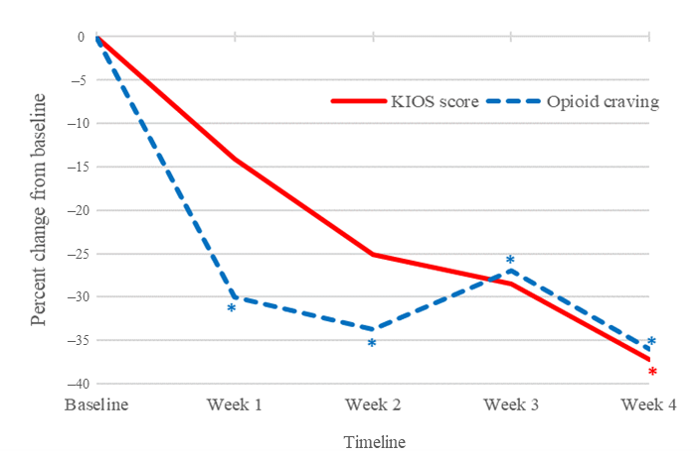
KIOS score and craving score. Craving scores were significantly lower at all time points after baseline (week 4; *P*=.013). The KIOS score, an index comprised of the sum of all assessment variables except for craving, was also significantly lower (week 4; *P*=.006).

**Figure 2:**
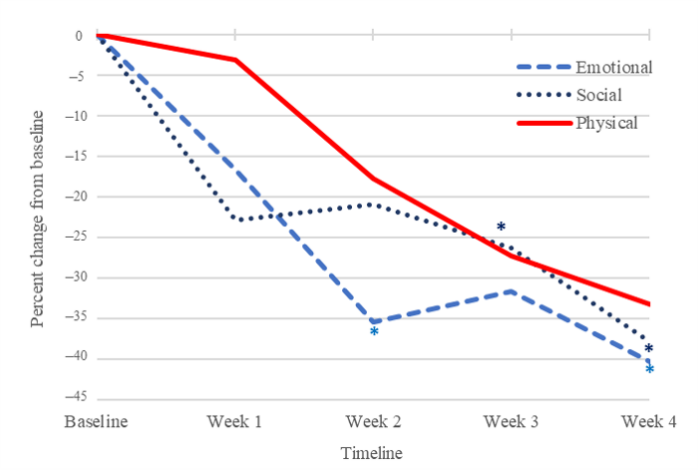
KIOS subscales. Emotional (week 4; *P*=.012) and social (week 4; *P*=.02) subscales were significantly lower compared to baseline at the end of the trial.

Figures 1 and 2 demonstrate how KIOS data can be used to track patient reporting over time. In Figure 1, the KIOS score and craving score were highly correlated, indicating that the symptom-reporting scales appear to be closely related to self-reported craving scores over time (Pearson *r*=0.550; *P*<.001). Two KIOS subscales were significantly lower at the end of the trial. Emotional (week 4; *P*=.01) and social (week 4; *P*=.02) subscales were significantly lower compared to the baseline (Figure 2).

## Discussion

### Principal Findings

The KIOS app is an adjunctive tool to OUD treatment that gives patients 24/7 access to behavioral intervention strategies and supportive and reinforcing behavioral guidance that potentially could augment and improve response to routine clinic-based counseling interventions. In this study, participants rated the primary outcomes of acceptability, usability, and utility very highly. The scores on the SUS-modified were in the excellent range. The mean response to all KIOS questionnaire data was 6.32 out of a maximum of 7.0 (SD 0.25), indicating an overall strong agreement. Participants used KIOS on average about 3 times weekly, which is a very good indication of sufficient use of the app to generate relevant advice.

### KIOS Acceptability

Participants on average used KIOS 3 times per week, which is the minimum requested in the study. This allowed sufficient time for relevant behavioral change between self-reports to generate pertinent recovery advice to the user. However, this may indicate a need to make the KIOS app more engaging, since participants could use the app daily if desired. Focusing the content on participants earlier in OUD treatment who may want more recovery advice or improving the experience by augmenting personalization could make the app more engaging [33,34]. Revisions to the app will include more explicit recommendations to continue in OUD treatment and more varied suggestions about mindfulness-oriented and positive self-care activities. Patients commented favorably on the individualized and specific nature of the advice and how this motivated and kept them on track in recovery. Some even commented that it seemed a counselor was responding to their self-report assessments in real time. This sense of copresence, of someone concurrently participating in the intervention with a psychological connection to the app user, was associated with improved satisfaction and efficacy in studies of other software apps [35,36].

### KIOS Usability

The SUS-Modified is a well-validated instrument [23,24]. KIOS was rated as very usable for these patients who were recruited from a community-based methadone maintenance treatment program. One item stood out as less positive: how cumbersome the app was to use. Since ease of use is clearly important, revisions to the app will involve attention to this aspect of use (eg, ease of moving between the home screen and different app functions). The revised KIOS app will be used on a smartphone platform to improve this aspect of usability.

### KIOS Utility

Utility was measured by a questionnaire specifically developed to evaluate the KIOS app. Participants strongly agreed on most questions related to usefulness, applicability, and relevance to recovery. Items that were less strongly endorsed were related to identifying personal triggers and improved health status. Since about one-third of the participants had been in treatment over 12 months, it is possible that identifying personal triggers was less of a treatment concern, and therefore, the mean score for this item was somewhat lower. In addition, since the study was only 4 weeks in duration, there was not much time to notice changes in general health status. Self-report of drug and alcohol use was modest in this sample, where only a minority of the participants was in treatment for less than 6 months. Much of the content of the app is aimed at changes in symptoms related to substance use problems; yet, participants who had minimal drug use problems and few mental health concerns rated the experience as helpful and relevant to their recovery. The randomized controlled study of KIOS will have a duration of 6 months and only focus on patients who are early in the treatment process. This will result in greater changes in drug use behavior and a longer period of time to capture potential changes in personal triggers and general health status. Revisions to the app will include more focus on positive behavioral changes to improve patient self-management and social support for recovery.

### KIOS Assessment Self-Reports

The scores derived from the KIOS self-report generated potentially useful data for both the KIOS user and the treatment team. As expected, the KIOS score and craving score were highly correlated. The correlation between these 2 measures suggests that the assessment variables, which collectively form the KIOS score capture relevant emotional, social, and physical phenomena that correspond with self-reported craving. Figures 1 and 2 demonstrate how KIOS data can usefully track patient reporting over time. These results could also be reviewed by the patient’s counselor to reinforce behavioral treatment planning and maximize the limited counseling time available at each appointment to assist the patient with this aspect of their care. The KIOS journal feature is available to help the patient record and organize pertinent thoughts and reflections, and the behavioral checklist helps to organize and track drug use and positive behaviors associated with improved recovery outcomes.

KIOS advice is designed to track patient self-report items related to OUD recovery over time. The KIOS app modifies the advice given to the user based on the person’s changes in self-report at each assessment. Advice is then directed specifically to the symptoms of most concern to the user. KIOS can draw the patient’s attention to triggers and potential problem behaviors with helpful and supportive advice in real time to reinforce recovery activities and goals at times of vulnerability to drug use. It may advise reaching out to support people or treatment providers depending on the type of a variable or increasing intensity of symptom reporting (eg, for increasing depression and insomnia consulting a medical provider), further encouraging integrated use of comprehensive treatment resources. It gives specific, evidence-informed suggestions for various symptoms including sleep hygiene, stress management, avoiding triggers, and pain reduction behaviors.

Expanded access to digital behavioral interventions has the potential to bridge a significant treatment gap due to the lack of counseling resources in many OUD treatment settings. However, there are very few digital behavioral apps that specifically address OUD [13,17,37,38]. Due to the nearly uniformly serious nature of this disorder, it is unlikely that a digital app by itself would be potent enough to help a person manage this disorder without professional treatment participation. The available apps focus primarily on connecting users to treatment services and peer support, making available CBT educational modules, using self-report check-ins, and using contingency management interventions. Their use has demonstrated significant improvements in some treatment outcomes [39]. For example, studies of reSET-O showed efficacy regarding opioid use or abstinence and in reinforcing and increasing treatment activity frequency in controlled trials [40]. The web-based CBT4CBT app improved retention and reduced drug use when combined with office-based treatment as usual [41]. It is one of the few digital interventions that has shown improved efficacy compared to in-person CBT in randomized controlled trials and demonstrates the potential power of these types of treatment augmentations [41,42]. The results of a randomized controlled trial of the A-CHESS digital app for use as an adjunct treatment in OUD did not demonstrate significant benefits compared to the control condition [16,43]. However, some results indicated that for specific subpopulations there could be benefits.

Other apps use a therapeutic relational approach using a conversational agent, such as those using a chatbot platform [12,44]. Some of these interventions have demonstrated efficacy [45]. They are currently used to help patients with a variety of mental health issues, although few have an evidence base [13,45-47]. The use of the W-SUDs modification of the Woebot app [12,13] has been associated with self-reported reductions in drug use and urges to use drugs and improvement in anxiety in users with substance use concerns [13]. The Woebot gives the impression of a therapeutic coach in the conversation generated by the chatbot. There is evidence from prior studies that some users are more likely to disclose information [48] to nonhuman apps, and that a strong therapeutic alliance can form [49-51]. Although not measured in this study, some participants commented on how KIOS responses seemed like real-time exchanges with a therapist. Future studies of KIOS should examine this aspect of the UI.

The study limitations include the short study duration in this trial to primarily test the acceptability, utility, and usability of the software. There was no control group, and it was not compared to another treatment condition or substance use treatment app to determine the specific benefits of KIOS compared to other apps or interventions for persons with substance use problems. The small sample size limits generalizability in several ways: to other opioid agonist treatment clinics or office-based OUD treatment, other patient samples that may be less interested in using digital apps, and in the ability to detect statistical differences between study variables. The patient focus group comments reflected the views of persons who were interested in using the app and potentially predisposed to having a positive user experience. KIOS relies on patient self-reporting to generate advice, so if a patient did not report accurate information, then they would not get accurate advice. This could be a problem if the patient’s counselor had access to the patient’s data. For example, if the patient was not ready to reveal their substance use, they might not give an accurate self-report. This should also be examined in future studies of the app.

### Conclusions

The KIOS app offers a different approach to the digital app for augmenting outpatient treatment for OUD. Potentially, the advice generated from the app may be more specific to the user because self-reported symptom clusters are used to identify appropriate behavioral interventions selected from evidence-based literature. It also has the potential advantage of using the therapeutic relational coaching approach that many users find more engaging.

Current plans for the development of KIOS include adding varied advice features, incorporating and reinforcing more positive recovery behaviors (eg, positive social support), and pursuing Food and Drug Administration approval as a prescription medical device. Although KIOS was studied in methadone maintenance clinics, it may be a very helpful tool in office-based buprenorphine addiction medicine treatment settings that often also have limited counseling availability. A National Institute on Drug Abuse–funded randomized controlled trial of KIOS is currently being conducted to evaluate its efficacy.

## Abbreviations

CBT: cognitive behavioral therapy
CMS: Community Medical Services
OUD: opioid use disorder
SUS: System Usability Scale
UI: user interface

## Data Availability

The data sets generated and analyzed during this study are not publicly available due to the proprietary nature of the device. Reasonable requests from the KIOS inventor, HRP, should be addressed to him specifically.

## References

[1] Centers for Disease Control and Prevention.

[2] American Psychiatric Publishing (2022). DSM-5-TR. 5th Edition.

[3] Ayanga D, Shorter D, Kosten TR (2016). Update on pharmacotherapy for treatment of opioid use disorder. Expert Opin Pharmacother.

[4] Substance Abuse and Mental Health Services Administration.

[5] Rice D, Corace K, Wolfe D, Esmaeilisaraji L, Michaud A, Grima A, Austin B, Douma R, Barbeau P, Butler C, Willows M, Poulin PA, Sproule BA, Porath A, Garber G, Taha S, Garner G, Skidmore B, Moher D, Thavorn K, Hutton B (2020). Evaluating comparative effectiveness of psychosocial interventions adjunctive to opioid agonist therapy for opioid use disorder: a systematic review with network meta-analyses. PLoS One.

[6] King VL, Brooner RK (2008). Improving treatment engagement in opioid-dependent outpatients with a motivated stepped-care adaptive treatment model. Jt Comm J Qual Patient Saf.

[7] Behavioral health workforce projections 2017-2030. Human Resources and Services Administration.

[8] Langdon KJ, Scherzer C, Ramsey S, Carey K, Rich J, Ranney ML (2021). Feasibility and acceptability of a digital health intervention to promote engagement in and adherence to medication for opioid use disorder. J Subst Abuse Treat.

[9] Riley WT, Oh A, Aklin WM, Wolff-Hughes DL (2019). National institutes of health support of digital health behavior research. Health Educ Behav.

[10] Aklin WM, Walton KM, Antkowiak P (2021). Digital therapeutics for substance use disorders: research priorities and clinical validation. Drug Alcohol Depend.

[11] Marsch LA, Campbell A, Campbell C, Chen CH, Ertin E, Ghitza U, Lambert-Harris C, Hassanpour S, Holtyn AF, Hser YI, Jacobs P, Klausner JD, Lemley S, Kotz D, Meier A, McLeman B, McNeely J, Mishra V, Mooney L, Nunes E, Stafylis C, Stanger C, Saunders E, Subramaniam G, Young S (2020). The application of digital health to the assessment and treatment of substance use disorders: the past, current, and future role of the national drug abuse treatment clinical trials network. J Subst Abuse Treat.

[12] Fitzpatrick KK, Darcy A, Vierhile M (2017). Delivering cognitive behavior therapy to young adults with symptoms of depression and anxiety using a fully automated conversational agent (Woebot): a randomized controlled trial. JMIR Ment Health.

[13] Prochaska JJ, Vogel EA, Chieng A, Kendra M, Baiocchi M, Pajarito S, Robinson A (2021). A therapeutic relational agent for reducing problematic substance use (Woebot): development and usability study. J Med Internet Res.

[14] (2018). Pear Therapeutics Inc. US Food and Drug Administration.

[15] Velez FF, Colman S, Kauffman L, Ruetsch C, Anastassopoulos K (2021). Real-world reduction in healthcare resource utilization following treatment of opioid use disorder with reSET-O, a novel prescription digital therapeutic. Expert Rev Pharmacoecon Outcomes Res.

[16] Gustafson DH, Landucci G, Vjorn OJ, Gicquelais RE, Goldberg SB, Johnston DC, Curtin JJ, Bailey GL, Shah DV, Pe-Romashko K, Gustafson DH (2024). Effects of bundling medication for opioid use disorder with an mhealth intervention targeting addiction: a randomized clinical trial. Am J Psychiatry.

[17] Nuamah J, Mehta R, Sasangohar F (2020). Technologies for opioid use disorder management: mobile app search and scoping review. JMIR Mhealth Uhealth.

[18] Kiburi SK, Ngarachu E, Tomita A, Paruk S, Chiliza B (2023). Digital interventions for opioid use disorder treatment: a systematic review of randomized controlled trials. J Subst Abuse Treat.

[19] Brezing CA, Brixner DI (2022). The rise of prescription digital therapeutics in behavioral health. Adv Ther.

[20] Hodges J, Waselewski M, Harrington W, Franklin T, Schorling K, Huynh J, Tabackman A, Otero K, Ingersoll K, Tiouririne NAD, Flickinger T, Dillingham R (2022). Six-month outcomes of the HOPE smartphone application designed to support treatment with medications for opioid use disorder and piloted during an early statewide COVID-19 lockdown. Addict Sci Clin Pract.

[21] Bowden CL, Priesmeyer R, Tohen M, Singh V, Calabrese JR, Ketter T, Nierenberg A, Thase ME, Siegel G, Siegel LH, Mintz J, El-Mallakh RS, McElroy SL, Martinez M (2021). Development of a patient-centered software system to facilitate effective management of bipolar disorder. Psychopharmacol Bull.

[22] Beyer H, Holtzblatt K The encyclopedia of human-computer interaction, 2nd Edition. Interaction Design Foundation.

[23] Bangor A, Kortum PT, Miller JT (2008). An empirical evaluation of the System Usability Scale. Int J Hum-Comput Int.

[24] Bangor A, Kortum PT, Miller JT (2009). Determining what individual SUS scores mean: adding an adjective rating scale. J Usability Stud.

[25] Kakko J, Alho H, Baldacchino A, Molina R, Nava FA, Shaya G (2019). Craving in opioid use disorder: from neurobiology to clinical practice. Front Psychiatry.

[26] McHugh RK, Fitzmaurice GM, Carroll KM, Griffin ML, Hill KP, Wasan AD, Weiss RD (2014). Assessing craving and its relationship to subsequent prescription opioid use among treatment-seeking prescription opioid dependent patients. Drug Alcohol Depend.

[27] Tsui JI, Anderson BJ, Strong DR, Stein MD (2014). Craving predicts opioid use in opioid-dependent patients initiating buprenorphine treatment: a longitudinal study. Am J Drug Alcohol Abuse.

[28] Weiss RD, Potter JS, Provost SE, Huang Z, Jacobs P, Hasson A, Lindblad R, Connery HS, Prather K, Ling W (2010). A multi-site, two-phase, Prescription Opioid Addiction Treatment Study (POATS): rationale, design, and methodology. Contemp Clin Trials.

[29] Lee JD, Nunes EV, Novo P, Bachrach K, Bailey GL, Bhatt S, Farkas S, Fishman M, Gauthier P, Hodgkins CC, King J, Lindblad R, Liu D, Matthews AG, May J, Peavy KM, Ross S, Salazar D, Schkolnik P, Shmueli-Blumberg D, Stablein D, Subramaniam G, Rotrosen J (2018). Comparative effectiveness of extended-release naltrexone versus buprenorphine-naloxone for opioid relapse prevention (X:BOT): a multicentre, open-label, randomised controlled trial. Lancet.

[30] Sinha IP, Smyth RL, Williamson PR (2011). Using the delphi technique to determine which outcomes to measure in clinical trials: recommendations for the future based on a systematic review of existing studies. PLoS Med.

[31] Abraham C, Michie S (2008). A taxonomy of behavior change techniques used in interventions. Health Psychol.

[32] Michie S, Richardson M, Johnston M, Abraham C, Francis J, Hardeman W, Eccles MP, Cane J, Wood CE (2013). The behavior change technique taxonomy (v1) of 93 hierarchically clustered techniques: building an international consensus for the reporting of behavior change interventions. Ann Behav Med.

[33] Forbes A, Keleher MR, Venditto M, DiBiasi F (2023). Assessing patient adherence to and engagement with digital interventions for depression in clinical trials: systematic literature review. J Med Internet Res.

[34] Borghouts J, Eikey E, Mark G, De Leon C, Schueller SM, Schneider M, Stadnick N, Zheng K, Mukamel D, Sorkin DH (2021). Barriers to and facilitators of user engagement with digital mental health interventions: systematic review. J Med Internet Res.

[35] Bulu ST (2012). Place presence, social presence, co-presence, and satisfaction in virtual worlds. Comput Educ.

[36] LaBrie JW, de Rutte JL, Boyle SC, Tan CN, Earle AM (2019). Leveraging copresence to increase the effectiveness of gamified personalized normative feedback. Addict Behav.

[37] Staiger PK, O'Donnell R, Liknaitzky P, Bush R, Milward J (2020). Mobile apps to reduce tobacco, alcohol, and illicit drug use: systematic review of the first decade. J Med Internet Res.

[38] Moore BA, Buono FD, Lloyd DP, Printz DM, Fiellin DA, Barry DT (2019). A randomized clinical trial of the recovery line among methadone treatment patients with ongoing illicit drug use. J Subst Abuse Treat.

[39] Fluetsch N, Whittington MD, Tice JA, Zapata LV, Mendola N, Chapman R, Campbell J, Pearson SD, Bradt P (2020). Digital health technologies as an adjunct to medication assisted therapy for opioid use disorder; final evidence report. Institute for Clinical and Economic Review.

[40] Maricich YA, Xiong X, Gerwien R, Kuo A, Velez F, Imbert B, Boyer K, Luderer HF, Braun S, Williams K (2021). Real-world evidence for a prescription digital therapeutic to treat opioid use disorder. Curr Med Res Opin.

[41] Shi JM, Henry SP, Dwy SL, Orazietti SA, Carroll KM (2019). Randomized pilot trial of web-based cognitive-behavioral therapy adapted for use in office-based buprenorphine maintenance. Subst Abus.

[42] Kiluk BD, Nich C, Buck MB, Devore KA, Frankforter TL, LaPaglia DM, Muvvala SB, Carroll KM (2018). Randomized clinical trial of computerized and clinician-delivered CBT in comparison with standard outpatient treatment for substance use disorders: primary within-treatment and follow-up outcomes. Am J Psychiatry.

[43] Gustafson DH, Landucci G, McTavish F, Kornfield R, Johnson RA, Mares ML, Westergaard RP, Quanbeck A, Alagoz E, Pe-Romashko K, Thomas C, Shah D (2016). The effect of bundling medication-assisted treatment for opioid addiction with mHealth: study protocol for a randomized clinical trial. Trials.

[44] Gardiner PM, McCue KD, Negash LM, Cheng T, White LF, Yinusa-Nyahkoon L, Jack BW, Bickmore TW (2017). Engaging women with an embodied conversational agent to deliver mindfulness and lifestyle recommendations: a feasibility randomized control trial. Patient Educ Couns.

[45] Gaffney H, Mansell W, Tai S (2019). Conversational agents in the treatment of mental health problems: mixed-method systematic review. JMIR Ment Health.

[46] Boumparis N, Schulte MHJ, Riper H (2019). Digital mental health for alcohol and substance use disorders. Curr Treat Options Psych.

[47] Laranjo L, Dunn AG, Tong HL, Kocaballi AB, Chen J, Bashir R, Surian D, Gallego B, Magrabi F, Lau AYS, Coiera E (2018). Conversational agents in healthcare: a systematic review. J Am Med Inform Assoc.

[48] Lucas GM, Gratch J, King A, Morency L (2014). It's only a computer: virtual humans increase willingness to disclose. Comput Hum Behav.

[49] Cook JE, Doyle C (2002). Working alliance in online therapy as compared to face-to-face therapy: preliminary results. Cyberpsychol Behav.

[50] Berry K, Salter A, Morris R, James S, Bucci S (2018). Assessing therapeutic alliance in the context of mHealth interventions for mental health problems: development of the mobile Agnew Relationship Measure (mARM) questionnaire. J Med Internet Res.

[51] Beatty C, Malik T, Meheli S, Sinha C (2022). Evaluating the therapeutic alliance with a free-text CBT conversational agent (Wysa): a mixed-methods study. Front Digit Health.

